# Optimal Planning Target Volume Margins for Elective Pelvic Lymphatic Radiotherapy in High-Risk Prostate Cancer Patients

**DOI:** 10.1155/2013/941269

**Published:** 2013-03-07

**Authors:** Benjamin K. Hinton, John B. Fiveash, Xingen Wu, Michael C. Dobelbower, Robert Y. Kim, Rojymon Jacob

**Affiliations:** Department of Radiation Oncology, University of Alabama at Birmingham, Hazelrig-Salter Radiation Oncology Center, Birmingham, AL 335294-0007, USA

## Abstract

*Purpose*. High-risk prostate cancer patients often receive radiotherapy (RT) to pelvic lymphatics (PLs). The aim of this study was to determine the safety margin around clinical target volume for PL (PL-CTV) to construct planning target volume for PL (PL-PTV) and for planning elective PL irradiation. *Methods and Materials*. Six patients who received RT to PL as part of prostate cancer treatment were identified. To determine average daily shifts of PL, the right and left IVs were contoured at 3 predetermined slices on the daily MV scans and their daily shifts were measured at these 3 levels using a measuring tool. *Results*. A total of 1,932 observations were made. Daily shifts of IV were random in distribution, and the largest observed shift was 13.6 mm in lateral and 15.4 mm in AP directions. The mean lateral and AP shifts of IV were 2.1 mm (±2.2) and 3.5 mm (±2.7), respectively. The data suggest that AP and lateral margins of 8.9 mm and 6.5 mm are necessary. *Conclusions*. With daily alignment to the prostate, we recommend an additional PL-CTV to PL-PTV conversion margin of 9 mm (AP) and 7 mm (lateral) to account for daily displacement of PL relative to the prostate.

## 1. Introduction

High-risk status in prostate cancer as defined by T stage, Gleason score, or presenting PSA confers a high probability of extraprostatic and lymphatic spread of cancer [1]. Radiotherapy (RT) in conjunction with neoadjuvant and concurrent long-term androgen suppression has been demonstrated to improve outcomes in such patients [2, 3]. The role of irradiating the pelvic lymphatics (PLs) is controversial [4, 5]. However, current NCCN guidelines suggest that patients with high-risk prostate cancer are candidates for RT to prostate, seminal vesicles, and PL.

Technological advances have allowed for higher doses to be delivered to the target, while minimizing the potential for interfraction variations which could result from positioning errors or organ motion [6–10]. During IGRT of high-risk prostate cancers, image guidance is accomplished by aligning the prostate contour with the daily image of the prostate. Though there is potential for day-to-day movement of the PL relative to the prostate, the PL field is not subjected to daily imaging for a number of reasons. Most RT institutions make use of radioopaque fiducials implanted within the prostate gland and daily kV orthogonal imaging for image guidance. Even when imaging systems such as cone-beam CT is available, the whole pelvis is not imaged daily as the procedure is time consuming and exposes the patient to unnecessary radiation. In addition, most image-guidance systems such as CBCT have field-size limitations, whereby the entire pelvic volume cannot be encompassed for daily imaging. Finally, it would be difficult to determine the ideal couch shifts when the prostate and PL show differing values of shifts on a particular day. The solution is to place an adequate margin around PL-CTV to create PL-PTV, whereby the PL-CTV will reliably lie with the PL-PTV when image guidance is performed to the prostate. This margin corresponds to the relative daily shift of PL to the prostate. In this study, we aimed to evaluate the daily shift of the iliac vessels (IVs), a surrogate for PL, relative to the prostate for determining the safety margin for contouring planning target volume (PL-PTV). We also aimed to dosimetrically validate the dose received by PL-CTV over the course of treatment with the addition of our recommended margin.

## 2. Methods and Materials

A cohort of six patients who received daily image-guided IMRT for high-risk prostate cancer on the Tomotherapy machine was randomly identified. All patients included in this study received RT to PL as part of their treatment, and their treatment courses were reviewed retrospectively. The pertinent clinical characteristics of these patients are summarized in Table 1. This research project was approved by the University of Alabama at Birmingham Institutional Review Board.

### 2.1. Radiotherapy Planning and Delivery

Each patient was simulated on a helical kV CT scanner with a full bladder and an empty rectum after immobilization in the supine position. Three mm thick simulation CT slices were generated, which were imported to the Eclipse planning system (Varian Medical Systems, Palo Alto, CA, USA) for target and OAR contouring. Given the unreliability of PL to be visualized on CT and the close proximity of PL to the IV, the IVs within the pelvis were used as surrogates for the PL in this study [11]. The IVs were contoured from the L5/S1 interspace to the level of the top of the femoral head. The PL clinical target volume (CTV) was contoured by adding a margin of 7 mm around the IV [12]. An additional margin varying between 7 mm to 10 mm according to physician preference was added around it to create PTV for treatment planning. The images were finally transferred to the TOMO (TomoTherapy Inc., Madison, WI, USA) workstation for radiotherapy planning. All patients were planned to receive 70, 56, and 50.4 Gy to the prostate, seminal vesicles, and PL, respectively, in 28 fractions using simultaneous integrated boost technique.

For treatment the initial alignment was performed by aligning the laser with skin marks on the patient. The entire PL volume was scanned daily, from the ischial tuberosities to the superior iliac crests, in all patients using megavoltage (MV) CT. The MV image of the day was matched with the planning digitally reconstructed radiograph (DRR), and correction vectors were calculated based upon alignment of the prostate. Daily couch shifts were applied prior to treatment delivery.

### 2.2. Interfraction Motion Analysis

**Figure 1:**
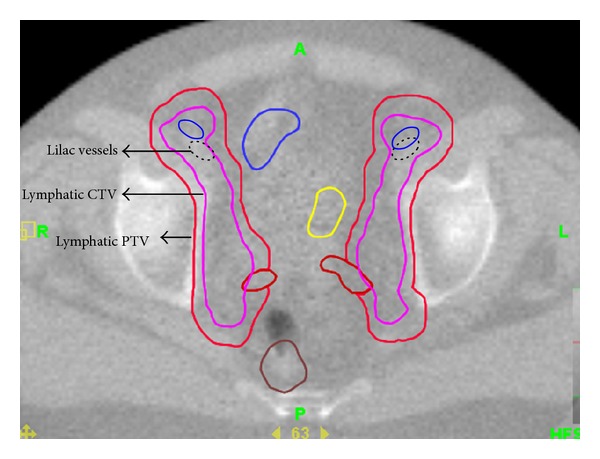
The figure demonstrates the location of the iliac vessels (IVs), PL-CTV, and PL-PTV on daily imaging. Original position of IV is shown using bold, and its location on a treatment day is shown using dotted line. Coverage of pelvic lymphatics is achieved despite shift in position of IV by adding sufficient margin around CTV to create PTV.

The interfraction motion analysis was performed retrospectively. We verified that the prostate on daily MV scan was fused appropriately to the treatment planning CT dataset. Care was taken to ensure that the IVs were visualized at each level to achieve consistency for each treatment day. To determine the average daily shifts of PL, the right and left IVs were contoured at three predetermined slices on the daily MV scans, namely, at the level of superior sacroiliac (SI) joints, inferior SI joints, and superior margin of femoral head. Anterior-Posterior (AP) and lateral shifts of IV were measured at these 3 levels after prostate fusion, using a measuring tool. AP and lateral shifts were measured at the most anterior lateral points of the IV by comparing the DRR to the daily MV image (Figure 1). The Tomotherapy Planned Adaptive software (TomoTherapy Inc., Madison, WI, USA) was used to analyze the fused images and measure daily shifts.

### 2.3. Shift Data Analysis

The absolute values of the shifts in the AP and lateral directions were collected for each patient. There were 12 data points corresponding to each treatment day at the three axial levels on the right and left side and two dimensions (AP and lateral). The mean shifts of IV (and standard deviations) were calculated using standard statistical methods. These data points were analyzed separately, according to their AP or lateral designations, and data points at the three axial levels were combined into these two groups.

### 2.4. Validation of Dosing

We dosimetrically assessed the recommended margins for the PL-CTV to PL-PTV conversion, by determining the dose received by the PL-CTV over the course of IGRT to the prostate. This was accomplished by analyzing data from three patients in the cohort for whom a sufficient margin (as recommended in this study) was added to PL-CTV to create PL-PTV during the original treatment planning. Using the Tomotherapy Planned Adaptive software, the PL-CTV contours were shifted manually on all slices to encompass the daily IV location with an uniform margin. Dose-volume information for PL-CTV was computed for each RT fraction, and cumulative RT doses over the entire course of RT were determined. The cumulative dose data were then compiled and compared to the prescription doses.

## 3. Results

A total of 1,932 observations were made, which included images from 28 and 26 treatment days for 3 and 2 patients, respectively. Images from 25 treatment days were studied in 1 patient. Images from 7 treatment days (all patients combined) were not used as the entire pelvis was not imaged on the day of treatment. The IVs were well visualized on the daily MV CT images despite the lower contrast.

**Figure 2:**
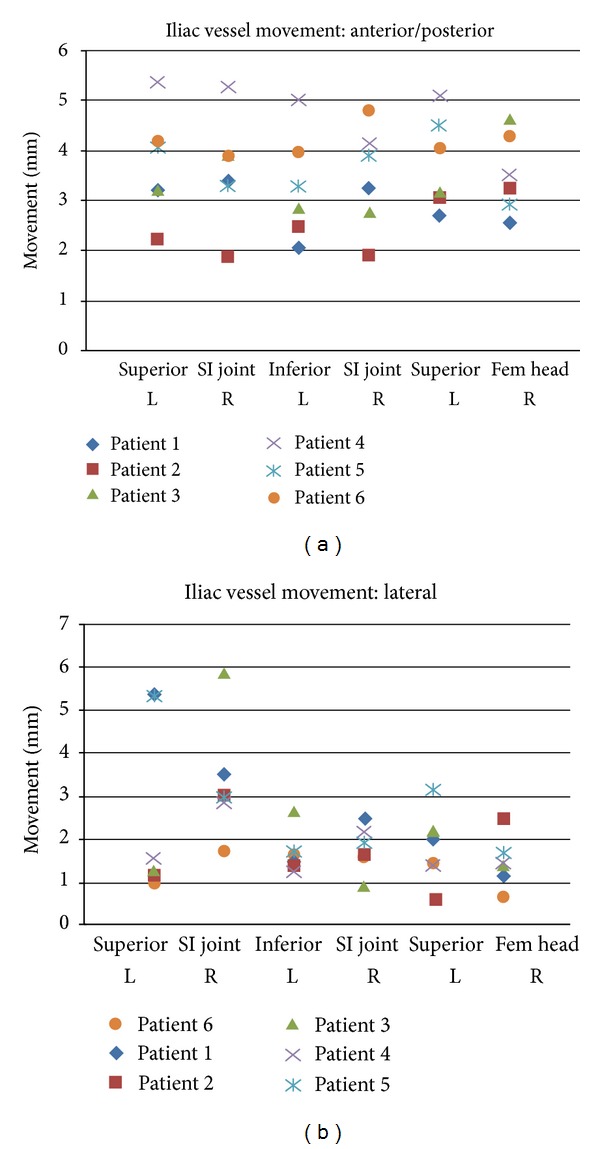
Illustration of the movement experienced by the iliac vessels relative to the prostate in 6 patients. The mean movement at 6 locations (3 axial levels, left, and right) is shown in the anterior-posterior (a) and lateral directions (b) for each patient.

**Figure 3:**
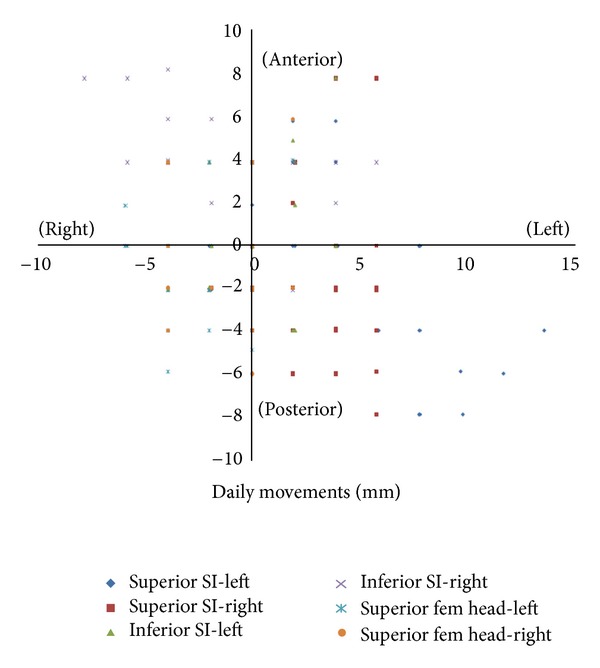
Movement data for a single patient. Movement data points of IV relative to their pretreatment location after image guided alignment to the prostate on each day of a 28-day treatment. This illustration represents a single patient data set based on all observations.

The daily shifts of IV were random in distribution; no systematic errors were observed. The largest observed shift in the lateral direction was 13.6 mm, and the largest observed shift in the AP direction was 15.4 mm in the AP directions. The mean lateral and AP shifts of IV were 2.1 mm (±2.2) and 3.5 mm (±2.7), respectively (Table 2). Figures 2(a) and 2(b) illustrate the movement experienced by the IV relative to the prostate in each patient. 95% of the observations lie within 8.9 mm and 6.5 mm in the AP and lateral directions. The daily movements of IV relative to their pretreatment location on each day of the 28-day treatment for a single patient are shown on Figure 3.

The dosimetric data of 3 patents in the cohort were analyzed for validation purposes. These 3 patients were originally planned with a PL-CTV to PL-PTV margin of 9 mm and 7 mm in the in the AP and lateral directions, respectively. A total of 84 dose-volume histograms were generated for this analysis. The mean dose to the CTV over the course of treatment was 54.3 Gy (±1.4) versus 50.4 Gy planned. The dose to CTV was 95% or more of planned doses during all 84 RT treatments (100%). Dose received by 90%, 95%, and 100% of the CTV (D90, D95, and D100) were 103.0% (±0.7%), 103.1% (±0.2%), and 103.3% (±0.4%) of the prescription dose, respectively. All dose parameters suggest adequate dosing of PL-CTV with the assigned CTV to PTV expansion (Table 3).

## 4. Discussion

The evolution of RT delivery modalities has allowed for greater confidence in daily localization of the target and delivery of high doses of radiation with improved accuracy and precision [13]. It is currently considered the standard of care to treat high-risk prostate cancers with a combination of androgen suppression and external beam radiotherapy. The role of pelvic lymphatic irradiation was evaluated in a randomized study by RTOG, with the conclusion that when AD is combined with whole pelvic radiation, the progression free survival in patients with high-risk of pelvic lymph node metastasis improved [4].

Conventionally, whole pelvic RT treats the prostate and PL to the same prescription dose (from 45 to 50.4 Gy), following which the boost RT is planned to the seminal vesicle and prostate. The use of high radiation dose to the prostate has been shown to improve biochemical control rates [14, 15]. Intensity-modulated RT (IMRT) allows delivery of higher “boost” doses to the prostate concurrently with whole pelvic RT, as opposed to escalating the dose afterward. This technique, called “simultaneous integrated boost (SIB)”, results in an overall shorter time period of treatment and reduces the dose to the rectum compared to sequential boosts [16].

The prostate is a nonrigid and deformable structure whose position varies depending on the bladder and rectal filling. RT dosage could be delivered more reliably to the prostate with the application of IGRT using B-mode acquisition and targeting (BAT), cone beam CT (CBCT), or, more recently, using intraprostatic fiducial markers [17–19]. However, dose delivery to the PL could vary depending on the position of the prostate to which image guidance is performed. Current consensus guidelines by genitourinary specialists recommend a radial CTV margin of 7 mm around iliac vessels for creation of PL-CTV [12]. This volume extends from the L5-S1 interspace to the top of the femoral heads and typically carves out the bowel, bladder, and bone. When shifts are performed to account for daily changes in prostate position, the PL-CTV will be subjected to the same movements applied to the prostate. Therefore, a sufficient CTV-PTV margin is necessary to account for daily anatomic variations. Caution should also be exercised during patient simulation to prevent systematic errors which may exceed the applied CTV-PTV margins [20]. This is usually achieved by proper immobilization, and sufficient bladder and bowel preparation to ensure that the anatomy during simulation is reasonably reproducible and approximates the typical appearance of these organs.

In this study, the IV shift data were collected in the AP and lateral axes on each treatment day within the three aforementioned slices on axial MV CT. Movements along the superior-inferior (SI) axis were not collected in this study, as we felt that these data would not significantly contribute to the formulation of CTV-PTV margin recommendations and would not be reportable at the same axial landmarks used for the AP and lateral directions. In addition, the longitudinal orientation of the IV in the pelvis decreases the potential for significant shift.

A potential limitation of this study lies in the fact that only three levels were used to characterize the course of the IV within the pelvis. It is reasonable to assume that a more exhaustive mapping of the IV would produce a more accurate picture of the daily location of the IV in relation to its pretreatment location, but we concluded that this effect would be minimal and would not likely affect the data in a significant way.

## 5. Conclusions

Currently, a consensus margin of 7 mm is used around IV to create PL-CTV for pelvic irradiation. With daily alignment to the prostate in IGRT, we recommend an additional CTV to PTV conversion margin of 9 mm (AP) and 7 mm (lateral) to account for daily displacement of PL relative to the prostate. Further, we have validated that these margins allow for sufficient dosing of the PL during IGRT directed to the prostate. 

## Figures and Tables

**Table 1:** Patient characteristics.

Patient number	Age (years)	T stage	Gleason Score	Presenting PSA ng/mL
1	55	T2c	3 + 3 = 6	112
2	76	T1c	3 + 4 = 7	25.8
3	76	T1c	4 + 4 = 8	10.6
4	72	T2a	4 + 3 = 7	15.4
5	71	T2b	3 + 3 = 6	27.9
6	67	T1c	3 + 3 = 6	20.1

Mean	71.5			22.95

**Table 2:** Mean values of the movement of the pelvic lymphatics during the course of treatment for all patients. Each value represents the mean shift at the three different levels of measurement.

Cohort	Left-iliac: lateral	Left-iliac: anterior-posterior	Right-iliac: lateral	Right-iliac: anterior-posterior
Mean	2.0 mm	3.6 mm	2.1 mm	3.5 mm
SD	±2.3	±2.7	±2.2	±2.6

**Table 3:** Dose-parameters for 3 patients who received radiation treatment with a CTV to PTV conversion margin of 9 mm (AP) and 7 mm (lateral) are shown. These data points are compared to planned doses received by 100% (D100), 95% (D95), and 90% (D90) of PTV.

	D100	D95	D90
	(as a percentage of prescription dose D100, 95, or 90)		
Patient 1	103.8	103.2	103.7
Patient 2	102.4	103.2	103.3
Patient 3	102.9	102.9	102.9
